# Patient-centered care - evidence in the context of professional health practice

**DOI:** 10.1590/0034-7167-2022-0448

**Published:** 2023-10-06

**Authors:** Josiane Bernart da Silva Ferla, Cristiano Miranda de Araujo, Marcos Herrerias de Oliveira, Luciana Branco Carnevale, Ana Paula Berberian

**Affiliations:** I Universidade Tuiuti do Paraná. Curitiba, Paraná, Brazil; II Instituto Federal de Educação Ciência e Tecnologia do Paraná. Curitiba, Paraná, Brazil; III Universidade Estadual do Centro-Oeste. Irati, Paraná, Brazil

**Keywords:** Patient-Centered Care, Person Centered Care, Health Personnel, Health Care Professional, Professional Practice, Cuidado Centrado no Paciente, Assistência Centrada no Paciente, Pessoal de Saúde, Profissional de Saúde, Prática Profissional, Atención Centrada en la Persona, Atención Dirigida al Paciente, Personal de Salud, Profesionales de la Salud, Práctica Profesional

## Abstract

**Objectives:**

to analyze patient-centered attitudes in care and sharing practices of nursing, speech therapy, dentistry and medicine professionals.

**Methods:**

cross-sectional research was used with 411 professionals, and the Patient-Practitioner Orientation Scale instrument was applied as a measure of outcome.

**Results:**

physicians presented higher mean scores, reflecting a patient-centered orientation, shared control, and focus on the person, with statistical difference for all domains (p<0.02). Dentists were the professionals who presented lower scores, especially in the sharing domain, with statistical difference in relation to nurses, speech therapists, and physicians (p<0.05).

**Conclusions:**

finally, the attitudes of professionals in the health areas studied indicated self-reported preference for centrality in patients. In this context, patient-centered care can be an important resource in health care when committed to overcoming the object man.

## RESUMO

**Objetivos:**

analisar as atitudes centradas no paciente nas práticas de cuidado e compartilhamento dos profissionais de enfermagem, fonoaudiologia, odontologia e medicina.

**Métodos:**

um estudo pesquisa transversal foi usado com 411 profissionais, e o instrumento Patient-Practitioner Orientation Scale foi aplicado como uma medida de resultado.

**Resultados:**

os médicos apresentaram escores médios mais elevados, refletindo orientação centrada no paciente, controle compartilhado e foco na pessoa, com diferença estatística para todos os domínios (p<0,02). Os dentistas foram os profissionais que apresentaram menores escores, principalmente no domínio compartilhamento, com diferença estatística em relação aos enfermeiros, fonoaudiólogos e médicos (p<0,05).

**Conclusões:**

por fim, as atitudes dos profissionais das áreas de saúde estudadas indicaram preferência autorreferida pela centralidade nos pacientes. Nesse contexto, o cuidado centrado no paciente pode ser um recurso importante na assistência à saúde quando comprometido com a superação do objeto homem.

## RESUMEN

**Objetivos:**

analizar las actitudes centradas en el paciente en el cuidado y compartir prácticas de profesionales de enfermería, logopedia, odontología y medicina.

**Métodos:**

se utilizó una investigación transversal con 411 profesionales, y como medida de resultado se aplicó el instrumento Patient-Practitioner Orientation Scale.

**Resultados:**

los médicos presentaron puntuaciones medias más altas, lo que refleja una orientación centrada en el paciente, control compartido y enfoque en la persona, con diferencia estadística para todos los dominios (p<0,02). Los odontólogos fueron los profesionales que presentaron puntajes más bajos, especialmente en el dominio compartir, con diferencia estadística en relación a los enfermeros, logopedas y médicos (p<0,05).

**Conclusiones:**

finalmente, las actitudes de los profesionales de las áreas de salud estudiadas indicaron preferencia autorreferida por la centralidad en los pacientes. En este contexto, el cuidado centrado en el paciente puede ser un recurso importante en el cuidado de la salud cuando se apuesta por la superación del objeto hombre.

## INTRODUCTION

Care, in the context of health, is generally related to carrying out procedures from a biological and medical perspective, reducing individuals’ health to physical and biological aspects, and impacting health professionals’ training and practice. When based on patterns established between normal and pathological, this practice compromises the implementation of approaches that recognize individuals and their health as biopsychosocially constituted^(1-4)^. In 1969, the hegemony of the biomedical model was greatly explored and, on this occasion, patient-centered care (PCC) begins to be described as a type of care committed to “understanding the patient as a single human being”, further mentioning that it should be placed at the heart of care, opposing principles and approaches pertinent to the biomedical model^(4)^.

In its Report of the Health Quality Committee in America, the Institute of Medicine (2001) considered PCC as a comprehensive part of the six pillars of quality health. According to the report, health care should be safe, effective, patient-centered, timely, efficient, and equitable. This document defines PCC as “respectful and responsive to the individual preferences, needs and values of the patient, and ensuring that patient values guide all clinical decisions”^(5)^. Studies also indicate that PCC has promoted important benefits for patients in what regards communication, greater satisfaction, and biomedical results^(3,6-7)^, in addition to contributing to the professional satisfaction of its providers^(8-13)^. From the involvement of both the patient and the different health professionals, the benefits of a patient-centered approach reiterate the relevance of the principles, actions, and procedures underlying it as well as the speed of assessment of its effects towards improved evidence-based health quality^(3,5,14-15)^.

Studies in which the Patient Practitioner Scale (PPOS)^(7)^ was used allowed to analyze patient-centered or disease-centered attitudes, in which the scores varied depending on the location, context, or professional education^(1-2,7,16-31)^. These studies usually indicate that patient-centeredness involves aspects such as greater importance attributed to patient participation in choices and decisions about their health care as well as the need to create a therapeutic relationship of balance of power between patients and professionals^(32)^. Studies recognize this orientation towards patient-centeredness as a determinant of these relationships, being relevant to better health care quality standards^(16-31)^, including Brazilian studies^(1-2,19)^.

On the premise that health processes are historically constituted, and that man should be conceived as the leading actor of this process, studies that address that PCC, including scoping reviews and World Health Organization (WHO) reports, contribute to overcoming technical barriers. Furthermore, these studies allow patients’ voice as well as the report of their needs, desires, and expectations to be advisors to their health care^(33-39)^, opposing functional and organic precepts based on a biomedical logic^(1,4,7)^.

Based on the above, this study aimed to analyze the centrality attitudes of nurses, speech therapists, dentists, and medical professionals in the Brazilian context, considering the care and sharing dimensions. Previous studies conducted in Brazil^(1-2,19)^ have focused on medical students, which justifies the development of a new study.

## OBJECTIVES

To analyze patient-centered attitudes in caring and sharing practices by nursing, speech therapy, dentistry, and medicine professionals.

## METHODS

### Ethical aspects

Data collection followed the procedures for research with human beings, and its release was approved by the research ethics committee, under CAAE (*Certificado de Apresentação para Apreciação Ética* - Certificate of Presentation for Ethical Consideration).

### Study design

The present study was reported according to the Strengthening the Reporting of Observational Studies in Epidemiology (STROBE)^(40)^.

This is a methodological and cross-sectional study, conducted from September to December 2020, throughout Brazil.

### Study population and eligibility criteria

A total of 411 health professionals in the areas of nursing, speech therapy, dentistry, and medicine participated in the study. Participants older than 18 years, who have completed a degree in health sciences in the areas of nursing, speech therapy, dentistry, and medicine, acting in direct patient care, in public or private care institutions, were included.

Sample recruitment was carried out using the snowball sampling technique^(41-43)^, which consists of a non-probabilistic sample form that uses reference chains. Data were collected individually from September to December 2020through the online research platform SurveyMonkey Audience^(44)^. For collection, there was no restriction of the respondent’s location, as long as the research remained within the Brazilian territory.

### Variables

After consenting to participate in the research, each health professional completed a sociodemographic questionnaire to characterize the sample. Subsequently, to analyze centrality attitudes, the translated version of the PPOS^(7)^ was also administered, with EOMP^(19)^ being the Brazilian Portuguese version. This instrument presents adequate values of internal consistency (Cronbach’s alpha = 0.605) and test-retest reliability (intraclass correlation coefficient = 0.670). The results obtained from this scale indicate whether a health professional has a more patient-centered or disease-centered orientation.

The scale comprises eighteen statements regarding two patient-related dimensions: sharing and caring. These statements must be classified on a six-point Likert scale, in which value 1 corresponds to “completely in agreement”, and value 6 to “completely at odds”. For all items, the highest values represent PCC, while the lowest values correspond to a physicianor disease-centered orientation. The authors of the original scale divide the total result into three groups: high (score ≥ 5.00, corresponding to a patient-centered orientation), medium (4.57 < score < 5.00), and low (score ≤ 4.57, corresponding to a diseaseor health professional-centered orientation). The results of the sharing and caring dimensions can be obtained, respectively, from the mean values of the nine items corresponding to each domain^(7)^.

PCC assessment scores were also analyzed through a sociodemographic questionnaire, considering possible confounding factors, such as age, gender, area of expertise, academic level, assisted subjects, level of care, and hospitalization experience.

To reduce the possible sources of bias, explanatory variables age, gender, area of expertise, academic level, assisted subjects, level of care, and hospitalization experience were investigated through a linear regression model, with the aim to assess their influence on the scores obtained by the questionnaires in the different areas.

### Statistical methods

The association between independent variables and the impact on the alteration of PPOS questionnaires’ scores were assessed considering the caring, sharing, and total domains. The scores for each domain were subjected to univariate analysis of variance (ANOVA). When ANOVA showed significance, a pair-to-peer comparison was performed using the post-hoc Tukey test. The variables that presented statistical significance in univariate analysis (α = 5%) were included in a multivariate linear regression model to assess the influence of these variables on the total score obtained. All analyses were performed using the software Statistical Package for the Social Sciences (SPSS)^(45)^ and Jamovi v.1.6^(46)^, adopting a significance level of 5%.

## RESULTS

Data involving a total of 503 participants were included in this study. However, 92 participants were excluded from the sample because they did not meet the eligibility criteria. Thus, the research had 411 participants (n = 411). The population’s mean age was 42 ± 10.4 years. Regarding gender, 13.7% were male and 86.3% female. Most participants were nurses, followed by speech therapists, dentists, and physicians, according to Table 1.

**Table 1:** Characteristics of the study population in the Brazilian national territory (n^*^=411), Brazil, 2020

Sociodemographic characteristics	n^*^ (%)
Gender	
Male	56 (13.7)
Female	352 (86.3)
Area of expertise	
Nursing	229 (55.8)
Speech therapy	84 (20.5)
Medicine	37 (9.0)
Dentistry	60 (14.1)
Academic level	
Undergraduate degree	42 (10.2)
Specialization	226 (55.1)
Master’s degree	75 (18.3)
Doctoral degree	54 (13.2)
Postdoctoral studies	13 (3.2)
Assisted subjects	
Children	251 (61.4)
Adolescents	234 (57.1)
Adults	337 (82.2)
Older adults	268 (65.4)
Level of care	
Primary	162 (39.5)
Secondary	185 (45.1)
Tertiary	186 (45.4)
Teaching	133 (32.6)
Hospitalization experience	
Staff	214 (52.7)
Family	377 (92.4)
Age in years - Mean (SD†)	42 (10.4)

* Number of participants; †SD - standard deviation.

Among the participants, 10.2% had an academic graduation level, 55.1% had at least one specialization in the area, 18.3% had a master’s degree, 13.2% had a doctoral degree, and 3.2% underwent postdoctoral internship. All data on the characteristics of the study population are available in Table 1.

Table 2 shows the situation, presenting the scores and their standard deviations for each individual question of the PPOS questionnaire.

**Table 2:** Scores by training areas and questions of the Patient Practitioner Scale^*^ in the Brazilian national territory, Brazil, 2020

PPOS^*^ Item [Mean (SD^†^)]	Nursing	Speech therapy	Dentistry	Medicine
1. Is it the healthcare professional who should decide what will be talked about in the consultation?	4.27 (1.7)	4.57 (1.77)	3.46 (1.63)	4.70 (1.43)
2. Although professional healthcare is more impersonal today, is this a small loss in exchange for advances in medicine?	3.97 (1.72)	4.17 (1.86)	3.47 (1.65)	4.72 (1.38)
3. Is physical examination the most important part of the consultation?	3.1 (1.63)	4.33 (1.80)	3.39 (1.69)	4.76 (1.32)
4. Is it generally best for patients that they do not have a complete explanation of their medical (health) condition?	5.51 (1.15)	5.28 (1.44)	5.69 (0.74)	5.59 (0.88)
5. Patients should rely on the knowledge of their health professionals and not try to find out about their condition for themselves.	3.44 (1.74)	3.95 (1.77)	3.33 (1.88)	4.19 (1.64)
6. When health professionals ask many questions about the patient’s history, are they intruding too much on personal issues?	5.79 (0.58)	5.92 (0.27)	5.94 (0.23)	5.78 (0.69)
7. If healthcare professionals are really good at diagnosis and treatment, the way they relate to patients is not that important.	5.72 (0.83)	5.83 (0.71)	5.72 (0.65)	5.87 (0.33)
8. Do many patients keep asking questions, even when they no longer have anything to learn in the consultation?	4.06 (1.65)	4.54 (1.52)	3.94 (1.58)	4.28 (1.46)
9. Should patients be treated as if they were partners of the health professional, with similar power, rights, and duties?	4.34 (1.71)	4.08 (1.81)	4.11 (1.82)	4.41 (1.72)
10. Do patients generally want to be reassured instead of having information about their health?	3.45 (1.47)	3.82 (1.70)	3.58 (1.42)	3.7 (1.24)
11. If the main characteristics of a health professional are to be sincere and friendly, will they not have much success?	5.14 (1.40)	5.2 (1.44)	5.28 (1.26)	5.46 (1.04)
12. When patients disagree with their healthcare professional, is it a sign that this professional does not have the respect and trust of their patient?	4.28 (1.48)	5.08 (1.20)	4.11 (1.65)	4.54 (1.31)
13. Can a treatment not succeed if it conflicts with the patient’s lifestyle or values?	5.28 (1.08)	5.15 (1.35)	5.17 (1.25)	5.24 (1.15)
14. Do most patients want to enter and leave the health professional’s office as soon as possible?	4.51 (1.37)	4.75 (1.39)	4.25 (1.48)	5.19 (0.99)
15. Should the patient always be aware that the healthcare professional is in charge?	4.53 (1.61)	4.53 (1.58)	3.11 (1.67)	4.72 (1.55)
16. Isn’t it so important to know the patient’s culture and history to treat their disease?	5.89 (0.58)	5.75 (0.79)	5.86 (0.42)	5.85 (0.35)
17. Is humor a major ingredient of the health professional in the treatment of the patient?	4.59 (1.47)	4.13 (1.53)	4.08 (1.56)	4.07 (1.49)
18. When the patient searches for information about their health status on their own, does this usually confuse more than help?	3.21 (1.62)	3.85 (1.55)	3.14 (1.64)	3.78 (1.64)
Full scale	4.49 (0.58)	4.66 (0.60)	4.30 (0.52)	4.77(0.56)

* Patient-Practitioner Orientation Scale;

† SD - standard deviation.

As can been seen, in Table 2, the score of full scale shows the following order: medicine, speech therapy, nursing and dentistry.

According to Table 3, the academic level variable differed significantly for the sharing domain and for the total scores, with lower scores for specialist professionals. The only independent variables that demonstrated statistical significance for the different domains of the PPOS questionnaire were area of expertise and academic level (p < 0.05). These professionals differed statistically from those with master’s and doctoral degrees (p < 0.05).

**Table 3:** Explanatory variables and comparison of mean scores for the Patient Practitioner Scale^*^ questionnaire in the Brazilian national territory, Brazil, 2020

Predictor variable	Category	Caring		Sharing		Total	
		Mean (SD^†^)	*p* value^‡^	Mean (SD^†^)	*p* value^‡^	Mean (SD^†^)	*p* value^‡^
Gender	Male	4.89(0.55)^a^	0.429	4.02(0.90)^a^	0.239	4.45(0.60)^a^	0.240
	Female	4.95(0.52)^a^		4.17(0.84)^a^		4.56(0.58)^a^	
Area of expertise	Nursing	4.89(0.54)^a^	0.001^§^	4.09(0.83)^a^	<.001^§^	4.49(0.58)^a^	<.001^§^
	Speech therapy	5.00(0.53)^abc^		4.33(0.84)^ac^		4.66(0.60)^ac^	
	Dentistry	4.83(0.47)^ac^		3.76(0.84)^b^		4.30(0.52)^b^	
	Medicine	5.16(0.43)^b^		4.38(0.84)^c^		4.77(0.56)^c^	
Academic level	Undergraduate degree	4.89(0.54)^a^	0.343	4.12(0.85)^ab^	0.001^§^	4.51(0.62)^ab^	0.009^§^
	Specialization	4.91(0.49)^a^		3.99(0.77)^b^		4.45(0.53)^b^	
	Master’s degree	5.05(0.49)^a^		4.34(0.89)^a^		4.69(0.58)^a^	
	Doctoral degree	4.99(0.58)^a^		4.38(0.86)^a^		4.69(0.63)^a^	
	Postdoctoral degree	4.88(0.83)^a^		4.69(1.11)^ab^		4.79(0.94)^ab^	
Assisted subjects (Y/N)	Children	4.99(0.52)	0.115	4.18(0.85)	0.431	4.59(0.59)	0.167
	Teens	4.97(0.54)	0.791	4.16(0.85)	0.437	4.56(0.60)	0.439
	Adults	4.94(0.54)	0.612	4.15(0.85)	0.620	4.54(0.59)	0.593
	Elderly	4.96(0.52)	0.297	4.20(0.80)	0.060	4.58(0.56)	0.071
Level of care (Y/N)	Primary	4.97(0.53)	0.324	4.18(0.86)	0.350	4.58(0.59)	0.254
	Secondary	4.91(0.49)	0.437	4.06(0.84)	0.141	4.48(0.56)	0.172
	Tertiary	4.96(0.52)	0.369	4.21(0.83)	0.184	4.59(0.58)	0.186
Hospitalization experience (Y/N)	Teaching	4.99(0.59)	0.260	4.22(0.95)	0.222	4.60(0.68)	0.193
	Staff	4.92(0.46)	0.264	4.12(0.85)	0.450	4.52(0.61)	0.277
	Family	4.96(0.52)	0.102	4.16(0.86)	0.576	4.55(0.59)	0.261

* Patient-Practitioner Orientation Scale;

† SD - standard deviation;

‡ p-value ANOVA test, Post-hoc - Tukey test; For variables with multiple correlations - different letters in the same column indicate statistical significance;

§ Significance level - p < 0.05

**Figure 1:**
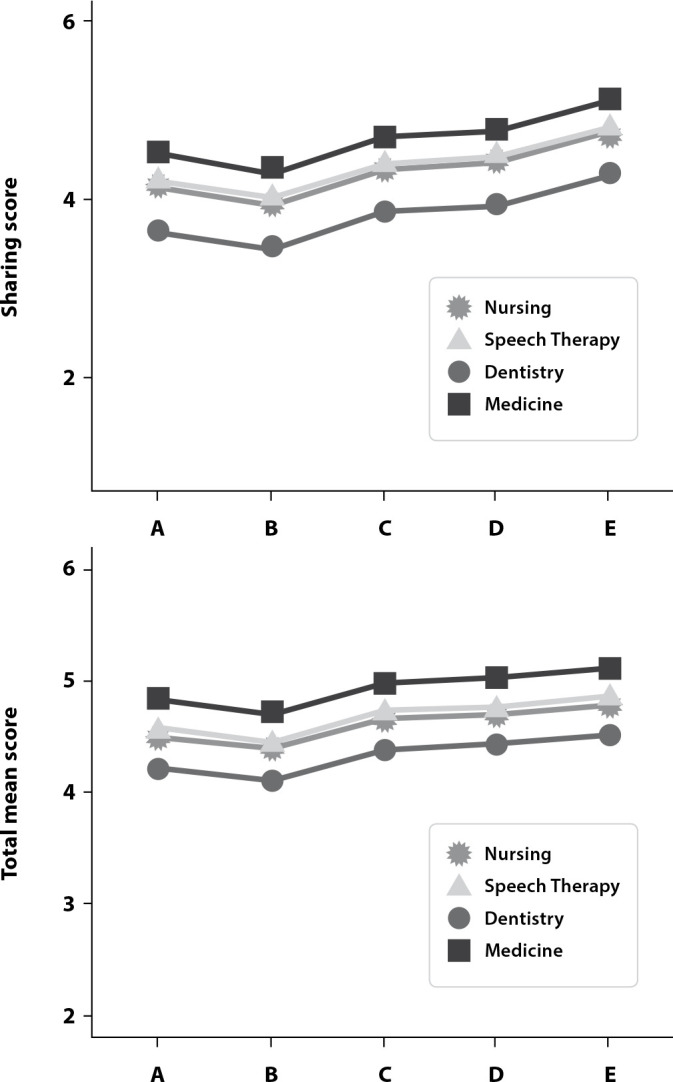
Interaction of the mean and observed values considering the academic level and area of expertise for the scores of sharing (a) and total (B)

Physicians presented higher mean scores, reflecting a patient-centered orientation, shared control, and focus on the person, with statistical difference for all domains (p < 0.02). Dentists were the professionals who presented lower scores, especially in the sharing domain, with statistical difference in relation to nurses, speech therapists, and physicians (p < 0.05). Increased academic level from specialization tends to increase PCC (Figures 1 (A) and 1 (B)).

When data were taken to a multivariate model, the area of expertise and academic level variables proved to be a significant predictor for the variation observed in the total scores of the PPOS questionnaire (p < 0.05). Moreover, the area of expertise variable was more important in the model than the academic level variable (Table 4).

**Table 4:** Predictor variables of the total score of the Patient Practitioner Scale^*^ questionnaire in the Brazilian national territory, Brazil, 2020

Domain	Predictor variable	β		Standard error	*p* value^†^
		Non-standardized	Standardized		
Score Total of the PPOS^*^	Constant	4.34		0.062	0.001
	Area of expertise	0.061	0.117	0.032	0.002^‡^
	Academic level	0.100	0.164	0.027	0.028^‡^

* Patient-Practitioner Orientation Scale; †p-value obtained by the multivariate linear regression model; ‡Significance level - p < 0.05

## DISCUSSION

This research found higher PCC scores for physicians and speech therapists than for nurses and dentists. The findings of this research on PPOS scores thus corroborate previous studies that indicated a self-reported preference for patient-centeredness. Among them, a multicenter study conducted in Portugal, India, and Iran^(20)^, with a sample composed of audiologists, demonstrated a significantly increased preference for patient-centered attitudes. The same occurred in investigations conducted in Saudi Arabia^(25)^, Sri Lanka^(16)^ and Spain^(17)^ with medical students, physicians, nurses and patients, respectively. In the present study, despite the statistical significance found when considering the area of expertise, the smaller sample size of the group of physicians and the use of non-probabilistic sampling decrease sample representativeness, requiring careful visualization of these findings.

In Brazil, three other studies were conducted using the same scale to assess centeredness attitudes. One of them was elaborated only for cultural adaptation and testing of psychometric properties for PPOS validity into Brazilian Portuguese^(19)^. The others, although performed with students^(1-2)^, corroborate our findings, pointing to more patient-centered attitudes.

As opposed to the present study, investigations carried out in Portugal^(27)^, with nurses, and in Kazakhstan^(28)^, with physicians and nurses, reveal that professionals have disease-centered attitudes based on a biomedical and care model. In the same sense, another study conducted before and after medical residency education points to a significant decline in patient-centered attitudes in male residents just after one year of residency^(22)^.

As for the variables that impacted the different assessment domains (caring, sharing), i.e., which presented a higher predictive factor for patient-centeredness, area of expertise and academic level stand out. It is important to highlight that the variation observed in the total scores of the PPOS questionnaire points to a greater importance of academic level in relation to area of expertise.

It is noteworthy that the medicine area presented higher scores in caring, sharing, and total, maintaining the highest score in 9 of the 18 items that make up the scale. These results indicate a trend of change from the dominant paradigm (biomedical) to a comprehensive view of both the care and the subject. Furthermore, aspects related to information sharing and decision-making factors as well as interpersonal relationships and professional-patient dialogue were listed among the issues most scored by this area. Likewise, a study conducted in China^(18)^ between 2019 and 2020 reported that the scores of the subscales of physicians in China pointed to a preference for patient-centeredness. In that study, the scores of subscales were higher in the caring domain and lower in the sharing domain. Moreover, the results for PPOS scores are essentially in agreement with our findings, although our scores were higher.

Regarding the area of speech therapy, research conducted in Portugal, Iran, and Iraq corroborates the findings of this study. This is because these authors present PPOS scores with self-reported preference for patient-centeredness in caring, sharing, and total. Although demonstrating a trend to patient-centeredness in speech therapy, the content of the items that show a lower score is consistent with traditionally implemented audiology practices, focusing on the application of diagnostic tests^(20)^.

Nursing and dentistry professionals had the lowest PPOS scores. Regarding dentistry, Madhan reports a score that points to disease-centeredness (3.38) and that gradually increases depending on the academic level^(30)^ as well as in our study.

With regard to nurses, our research points to average levels of patient-centered attitudes, revealing higher scores in caring to the detriment of sharing. Consistent with other studies^(7,34)^, all participants scored lower on the sharing than on the caring domain. According to the authors, this result may be because health professionals have a strong belief in patients’ emotional and psychosocial factors, but they are less supportive in sharing information and empowering patients in decision-making. Such inconsistency between the two scores may be due to the traditional domain of the biomedical model that still prevails in most clinical practice in nursing^(7)^. The central question of the biomedical model, in this sense, lies in the fact that it is too restricted in its explanatory power. This can be considered an obstacle to clinical practice, since it does not answer many questions related to the subject’s biopsychosocial aspects and the socioeconomic problems surrounding the disease^(32)^.

Regarding the academic level variable, the higher the level of education, the greater the impact on the scores, especially from specialization. Likewise, a study states that this educational progression related to professional experience can develop patient-centeredness in health professionals. As it rises academically, professionals seem more predisposed to sharing knowledge and information, which consequently contributes to patient empowerment regarding health-disease processes^(35)^.

Patient-centered attitudes are presented as mean, following PPOS scores. This is because none of the scores reached a value greater than or equal to 5.00 or a value lower than 4.30, as presented by dentistry. It is also noteworthy that the average score per explanatory variable was not lower than 3.76, also demonstrating a tendency of these health professionals to favor the focus on patient care to the detriment of sharing care with patients.

Patient-centeredness is an important determinant of health practices, which has its foundation in the precepts that guide the principle of comprehensiveness of care. It is thus closely related to patients’ results, such as greater satisfaction and treatment compliance, highlighting the social and historical determinants that involve the subject. This directly implies health promotion, interprofessional and collaborative practice, professional-patient relationships, dialogue that permeates health systems, and overall health quality and safety.

### Study limitations

With the study under a cross-sectional design, it was possible to observe that it could be more appropriate to analyze centrality of care practice in patients based on longitudinal drawings and in line with patients, in order to correlate, more accurately, both health professionals and patients within this model of care. Moreover, the use of non-probabilistic sampling does not eliminate the risk of confounding factors, regardless of sample size. The reduced sample size in the group of physicians reduces the representativeness of the assessed sample.

### Contributions to health

This study reports important notes on PCC in professional care, both nationally and internationally, as well as the conceptions that guide and determine ways of understanding and developing health practice.

However, it is important to emphasize that, although PCC is an important movement in relation to health practices in the search for overcoming the historically dominant biomedical model, nursing and dentistry professionals still tend to prioritize a care that is not very patient-centered to the detriment of medical and speech therapy professionals, pointing us that giving care a new meaning still timidly moves towards an effective paradigm shift.

## CONCLUSIONS

In the present study, we reported for the first time a complete description and comparison of self-reports of physicians, nurses, dentists, and speech therapists about patient-centeredness. We also estimated the mean scores of these professionals regarding the gender, area of expertise, academic level, assisted subjects, level of care, and hospitalization experience predictor variables, using a PPOS scale validated in caring, sharing and total. For these domains, the only independent variables that demonstrated statistical significance were academic level and area of expertise, the latter being of greater importance. It is noteworthy that physicians and speech therapists had the highest PCC scores, followed by nurses and dentists. However, these findings must be viewed with caution due to the reduced sample representativeness. PCC constitutes an important movement regarding health practices, committed to overcoming the historically dominant biomedical paradigm. It also consolidates conceptions and practices capable of conceiving and prioritizing beyond the disease, the social and historical determinants that involve the subject. Finally, we hope that this research can encourage future studies on PCC and health professionals.
